# Supplementary material for: Evaluation of Anthocyanin Profiling, Total Phenolic and Flavonoid Content, and Antioxidant Activity of Korean Rubus Accessions for Functional Food Applications and Breeding
Source: Antioxidants (Basel). 2025 Aug 18;14(8):1012. doi: 10.3390/antiox14081012 (PMC12382936; doi:10.3390/antiox14081012)
Supplement: Supplementary file 1 [file antioxidants-14-01012-s001.zip › antioxidants-3813415-supplementary.pdf]

Table S1. Radical scavenging activities in various *Rubus* berries.

A. DPPH scavenging activity

No.	Resources	**10 (μg/mL)**	**30 (μg/mL)**	**100 (μg/mL)**	**300 (μg/mL)**	**1000 (μg/mL)**	**IC$_{50}$ (μg/mL)**
1	V7	3.71 ±2.20^{ab}	18.04 ± 4.77^{bc}	49.48 ± 0.43^{g}	58.05 ± 1.87^{cde}	92.61 ± 0.55^{b}	112.15
2	Maple	9.10 ±3.19^{abc}	13.72 ± 3.72^{abc}	35.83 ± 1.17^{cdef}	55.95 ± 2.59^{cd}	91.75 ± 2.47^{b}	240.82
3	Blackpearl	6.28 ±2.40^{ab}	16.02 ± 1.13^{bc}	35.76 ± 3.06^{cdef}	56.49 ± 3.37^{cd}	93.48 ± 0.59^{b}	237.41
4	Resource A	9.98 ±3.31^{abc}	17.40 ± 3.35^{bc}	38.87 ± 1.19^{ef}	51.48 ± 3.47^{bc}	90.02 ± 2.88^{b}	276.58
5	Resource B	6.70 ±2.97^{abc}	17.00 ± 4.37^{bc}	29.91 ± 1.46^{bcd}	57.38 ± 1.75^{cde}	89.15 ± 3.00^{b}	246.25
6	Resource C	8.77 ±2.17^{abc}	19.86 ± 2.10^{c}	37.14 ± 2.29^{def}	59.98 ± 3.79^{def}	88.35 ± 3.24^{b}	212.61
7	Resource D	9.40 ±1.36^{abc}	15.50 ± 0.46^{abc}	40.51 ± 3.87^{ef}	67.81 ± 1.82^{f}	93.56 ± 0.22^{b}	169.52
8	Resource E	9.00 ±3.56^{abc}	18.00 ± 4.61^{bc}	43.93 ± 3.21^{fg}	65.50 ± 4.45^{ef}	93.87 ± 1.01^{b}	156.26
9	Resource F	20.00 ±4.09^{d}	41.38 ± 4.00^{d}	79.40 ± 4.53^{h}	93.96 ± 0.88^{g}	94.56 ± 0.40^{b}	45.88
10	Resource G	11.52 ±1.21^{bcd}	16.10 ± 2.82^{bc}	35.33 ± 4.77^{cde}	63.44 ± 2.05^{def}	92.88 ± 1.87^{b}	204.40
11	Resource H	15.60 ±3.67^{cd}	17.54 ± 2.46^{bc}	29.99 ± 4.26^{bcd}	46.57 ± 1.06^{b}	78.67 ± 2.51^{a}	374.85
12	Resource I	7.34 ±4.08^{abc}	10.23 ± 3.59^{ab}	25.80 ± 1.58^{b}	44.26 ± 1.10^{b}	77.46 ± 3.31^{a}	421.09
13	Resource J	2.09 ±2.20^{a}	8.96 ± 0.78^{ab}	28.09 ± 3.20^{bc}	63.26 ± 2.48^{def}	94.02 ± 0.36^{b}	224.61
14	Resource K	4.58 ±3.16^{ab}	9.83 ± 3.49^{ab}	26.46 ± 1.22^{b}	63.88 ± 1.72^{def}	94.56 ± 0.12^{b}	225.83
15	Resource N	4.10 ±3.91^{ab}	5.96 ± 1.45^{a}	16.79 ± 0.70^{a}	35.09 ± 4.59^{a}	77.11 ± 3.03^{a}	548.39

B. ABTS cation radical scavenging activity

No.	Resources	**10 (μg/mL)**	**30 (μg/mL)**	**100 (μg/mL)**	**300 (μg/mL)**	**1000 (μg/mL)**	**IC$_{50}$ (μg/mL)**
1	V7	13.68±1.16^{cd}	30.43±1.85^{d}	76.49±0.92^{g}	97.34±0.18^{def}	99.74±0.47^{ab}	59.74
2	Maple	12.93±0.92b^{cd}	24.17±1.97^{cd}	65.92±0.77^{ef}	97.14±0.18^{de}	100.51±0.64^{ab}	73.31
3	Blackpearl	7.37±3.98^{ab}	16.40±4.36^{abc}	51.45±1.68^{bc}	93.80±1.15^{c}	99.09±0.30^{a}	97.11
4	Resource A	6.79±0.36^{a}	17.35±5.94^{abc}	59.35±4.09^{de}	96.66±0.18^{d}	100.68±0.48^{b}	84.42
5	Resource B	8.18±1.94^{abc}	16.68±1.93^{abc}	47.47±0.97^{b}	91.39±1.28^{b}	99.63±0.18^{ab}	111.54
6	Resource C	11.77±1.37^{abcd}	26.15±3.40^{d}	69.04±2.50^{f}	98.81±0.34^{efg}	100.34±0.59^{ab}	68.93
7	Resource D	14.54±1.60^{d}	29.30±1.91^{d}	79.78±2.11^{g}	99.79±0.48^{g}	99.84±0.32^{ab}	60.52
8	Resource E	14.29±0.55^{d}	22.80±0.48^{bcd}	57.62±2.82^{cd}	100.21±0.18^{g}	100.48±0.84^{ab}	156.26
9	Resource F	58.64±2.46^{e}	82.88±2.54^{e}	99.89±0.18^{h}	100.00±0.32^{g}	99.74±0.48^{ab}	8.53
10	Resource G	16.65±1.89^{d}	28.25±5.06^{d}	64.39±0.64^{ef}	99.84±0.00^{g}	100.91±0.49^{b}	72.13
11	Resource H	16.61±1.63^{d}	27.43±1.13^{d}	61.55±0.48^{de}	93.05±0.36b^{c}	99.48±0.36^{ab}	76.31
12	Resource I	12.76±1.95^{bcd}	30.70±0.94^{d}	68.70±3.39^{f}	94.54±0.19^{c}	100.00±0.32^{ab}	65.55
13	Resource J	11.91±0.55^{abcd}	23.94±2.12^{cd}	64.63±3.53^{ef}	99.10±0.18^{fg}	99.41±0.49^{ab}	74.84
14	Resource K	7.50±1.36^{ab}	14.18±1.14^{ab}	47.31±1.14^{b}	92.94±0.19^{bc}	100.60±0.94^{ab}	111.78
15	Resource N	8.15±2.96^{abc}	13.75±2.04^{a}	32.61±2.36^{a}	72.88±1.36^{a}	99.89±0.38^{ab}	186.37

C. Superoxide radical scavenging activity

No.	Resources	**10 (μg/mL)**	**30 (μg/mL)**	**100 (μg/mL)**	**300 (μg/mL)**	**1000 (μg/mL)**	**IC$_{50}$ (μg/mL)**
1	V7	9.00±1.83^{ef}	13.80±1.83^{def}	38.40±0.69^{efg}	61.20±3.86^{de}	82.80±3.86^{bc}	201.75
2	Maple	8.66±1.89^{def}	13.81±3.57^{def}	43.92±1.89^{g}	65.77±1.43^{ef}	84.33±4.34^{bc}	155.66
3	Blackpearl	8.09±2.59^{def}	9.96±1.90^{d}	34.44±2.59^{def}	61.83±0.72^{de}	80.29±2.59^{bc}	213.64
4	Resource A	7.71±2.53^{def}	13.18±1.41^{de}	40.97±1.22^{fg}	61.05±1.22^{de}	88.44±3.22^{cd}	189.89
5	Resource B	2.03±2.07^{cde}	4.97±2.82^{bcd}	26.19±4.88^{cd}	58.92±0.78^{cde}	78.56±4.35^{bc}	245.51
6	Resource C	9.23±3.41^{ef}	13.30±2.97^{de}	41.20±1.97^{fg}	65.02±1.97^{ef}	85.84±3.41^{bcd}	173.87
7	Resource D	-5.87±3.69^{c}	0.49±4.48^{bc}	14.43±3.69^{b}	50.61±4.48^{b}	65.77±4.23^{a}	296.62
8	Resource E	-15.00±1.57^{b}	-2.95±4.09^{b}	1.36±2.84^{a}	27.95±4.17^{a}	65.91±2.36^{a}	706.60
9	Resource F	-28.31±4.12^{a}	-16.18±0.78^{a}	28.31±2.81^{cd}	53.48±2.70^{bc}	83.15±3.57^{bc}	272.33
10	Resource G	3.14±4.01^{def}	6.95±1.55^{cd}	25.34±1.35^{c}	58.97±1.35^{cde}	88.57±4.85^{cd}	246.67
11	Resource H	10.04±2.05^{efg}	19.42±3.41^{efg}	43.75±1.34^{g}	64.96±0.77^{ef}	108.04±3.54^{e}	158.94

12	Resource I	18.03±1.62^{g}	23.19±1.62^{g}	41.45±2.93^{fg}	59.95±1.41^{cde}	96.25±4.52^{d}	192.40
13	Resource J	11.27±2.82^{fg}	22.30±3.54^{fg}	43.66±3.73^{g}	69.25±0.81^{f}	85.68±1.63^{bc}	149.55
14	Resource K	0.48±2.99^{cd}	7.18±2.19^{cd}	32.54±1.44^{cde}	64.11±2.87^{ef}	87.56±0.83^{cd}	210.61
15	Resource N	6.80±1.96^{def}	9.92±4.50^{d}	26.35±3.93^{cd}	54.67±2.60^{bcd}	76.20±1.70^{ab}	267.01

D. Fe2+ chelating scavenging assay

No.	Resources	**10 (μg/mL)**	**30 (μg/mL)**	**100 (μg/mL)**	**300 (μg/mL)**	**1000 (μg/mL)**	**IC_{50} (μg/mL)**
1	V7	0.83±2.50^{a}	4.47±2.87^{a}	15.56±3.44^{cd}	43.38±3.44^{cd}	83.44±2.29^{cde}	415.68
2	Maple	-2.28±1.49^{a}	1.79±1.13^{a}	9.76±2.58^{abc}	37.72±1.13^{bc}	79.19±1.49^{bc}	507.28
3	Blackpearl	0.68±1.56^{a}	3.41±2.70^{a}	3.58±1.56^{a}	39.01±1.18^{bc}	87.22±1.02^{e}	459.56
4	Resource A	0.32±1.47^{a}	4.50±2.55^{a}	25.72±1.93^{e}	50.48±2.43^{e}	84.46±1.67^{cde}	296.11
5	Resource B	-1.49±4.69^{a}	1.65±3.75^{a}	14.55±4.13^{cd}	57.85±4.32^{f}	97.90±2.06^{f}	263.74
6	Resource C	-2.12±2.59^{a}	2.61±1.13^{a}	11.73±0.56^{c}	47.88±0.56^{de}	81.60±0.56^{bcd}	343.98
7	Resource D	2.01±2.53^{a}	5.18±2.01^{a}	12.04±2.09^{c}	38.80±3.62^{bc}	76.25±2.53^{b}	509.39
8	Resource E	-1.60±1.47^{a}	-0.16±2.00^{a}	4.49±0.56^{ab}	22.76±2.42^{a}	69.55±1.47^{a}	707.53
9	Resource F	-3.68±2.32^{a}	3.01±2.09^{a}	14.38±2.32^{cd}	57.19±2.90^{f}	112.21±3.07^{g}	266.40
10	Resource G	-2.57±2.43^{a}	1.29±0.56^{a}	11.09±0.56^{bc}	41.16±1.67^{bcd}	80.55±1.11^{bcd}	457.14
11	Resource H	2.77±4.26^{a}	3.58±2.99^{a}	12.21±3.70^{c}	34.53±4.26^{b}	82.41±2.93^{cde}	526.22
12	Resource I	-4.51±1.48^{a}	-0.48±0.97^{a}	9.82±0.56^{abc}	40.74±0.56^{bc}	81.96±0.56^{cde}	457.24
13	Resource J	-0.32±3.48^{a}	1.61±4.20^{a}	14.15±1.67^{cd}	41.64±1.93^{bcd}	82.80±2.01^{cde}	442.16
14	Resource K	-4.41±0.94^{a}	0.47±1.44^{a}	19.06±0.55^{de}	63.62±0.00^{f}	84.88±0.00^{de}	238.87
15	Resource N	-0.17±2.07^{a}	4.31±4.91^{a}	12.60±2.87^{cd}	36.32±3.59^{b}	87.40±2.07^{e}	487.49

Values denote mean ± standard deviation (n=3). Different superscripts in the same column indicate significant differences based on Tukey's test ($p < 0.05$).
